# *Megasphaera* in the Stool Microbiota Is Negatively Associated With Diarrheal Cryptosporidiosis

**DOI:** 10.1093/cid/ciab207

**Published:** 2021-05-04

**Authors:** Maureen A Carey, Gregory L Medlock, Masud Alam, Mamun Kabir, Md Jashim Uddin, Uma Nayak, Jason Papin, A S G Faruque, Rashidul Haque, William A Petri, Carol A Gilchrist

**Affiliations:** 1 Division of Infectious Diseases and International Health, Department of Medicine, University of Virginia, Charlottesville, Virginia, USA; 2 Department of Biomedical Engineering, University of Virginia, Charlottesville, Virginia, USA; 3 International Centre for Diarrhoeal Disease Research, Bangladesh; 4 Department of Public Health Sciences, University of Virginia, Charlottesville, Virginia, USA

**Keywords:** *Cryptosporidium*, microbiome/microbiota, parasite, diarrhea, Bangladesh

## Abstract

**Background:**

The protozoan parasites in the *Cryptosporidium* genus cause both acute diarrheal disease and subclinical (ie, nondiarrheal) disease. It is unclear if the microbiota can influence the manifestation of diarrhea during a *Cryptosporidium* infection.

**Methods:**

To characterize the role of the gut microbiota in diarrheal cryptosporidiosis, the microbiome composition of both diarrheal and surveillance *Cryptosporidium*-positive fecal samples from 72 infants was evaluated using 16S ribosomal RNA gene sequencing. Additionally, the microbiome composition prior to infection was examined to test whether a preexisting microbiome profile could influence the *Cryptosporidium* infection phenotype.

**Results:**

Fecal microbiome composition was associated with diarrheal symptoms at 2 timepoints. *Megasphaera* was significantly less abundant in diarrheal samples compared with subclinical samples at the time of *Cryptosporidium* detection (log_2_ [fold change] = –4.3; *P *= 10^–10^) and prior to infection (log_2_ [fold change] = –2.0; *P* = 10^–4^); this assigned sequence variant was detected in 8 children who had diarrhea and 30 children without diarrhea. Random forest classification also identified *Megasphaera* abundance in the pre- and postexposure microbiota as predictive of a subclinical infection.

**Conclusions:**

Microbiome composition broadly, and specifically low *Megasphaera* abundance, was associated with diarrheal symptoms prior to and at the time of *Cryptosporidium* detection. This observation suggests that the gut microenvironment may play a role in determining the severity of a *Cryptosporidium* infection.

**Clinical Trials Registration**. NCT02764918.

## Teaser

*Megasphaera* abundance in the stool of Bangladeshi infants is associated with the development of diarrhea upon infection with the *Cryptosporidium* parasite.

Protozoan parasites in the *Cryptosporidium* genus cause both acute diarrhea and subclinical (ie, nondiarrheal) disease, and both clinical outcomes are associated with poor physical and neurocognitive growth in infants [1–6]. These parasites are the fifth leading cause of diarrhea in young children [7], and recent studies have estimated the global burden of *Cryptosporidium* diarrhea mortality to be as high as 50 000 deaths annually [8]. This burden is disproportionately borne by young children [9]. Importantly, no therapies exist to treat *Cryptosporidium* infection in children or immunocompromised individuals [10]. Thus, there is a pressing need to prevent cryptosporidiosis mortality.

Understanding the difference in the host, parasite, and environment during acute diarrheal and subclinical infections may reveal new therapeutic solutions. Human polymorphisms are associated with an increased host susceptibility to cryptosporidiosis; however, these mutations do not completely explain the differences in infection outcomes [11, 12]. Parasite genetics (within and across species) have been associated with differences in their host range [13–16]. The role of the microbiome upon infection by *Cryptosporidium* has been examined in healthy adults [17] and animals [18, 19]; however, its role in differentiating diarrheal and subclinical infections is not known, nor is the impact of any differences in the microbiome composition occurring during infant cryptosporidiosis.

Here, we interrogate the association between diarrheal status during cryptosporidiosis and a child’s microbiome using fecal samples from infants living in Mirpur and Mirzapur, Bangladesh. In Mirpur, *Cryptosporidium* diarrhea was frequent (24% of infections); detected *Cryptosporidium* species included *Cryptosporidium hominis*, *Cryptosporidium parvum*, and *Cryptosporidium meleagridis*, with *C. hominis* as the most common. In contrast, most infections in Mirzapur were subclinical (98%), and *C. meleagridis* was the most common detected species [1]. Because *Cryptosporidium*-associated diarrhea was infrequent in Mirzapur and most infections involved *C. meleagridis* rather than *C. hominis* or *C. parvum*, the association between diarrheal status and microbiome composition in infants in Mirzapur could not be decoupled from an alternative infection phenotype caused by *C. meleagridis*. We therefore focused our analysis on Mirpur due to the variation in diarrheal status and the dominance of the *C. hominis* species in this population. We found that the microbiota demonstrated high variability between children but, despite this observation, microbiota composition and a low abundance of *Megasphaera* were associated with diarrheal symptoms both at the time of *Cryptosporidium* detection and prior to infection. Thus, we propose that *Megasphaera* may prevent acute diarrhea during parasite infection or at least can serve as a biomarker for other unknown protective factors.

## MATERIALS AND METHODS

### Cohort

**Figure 1.**
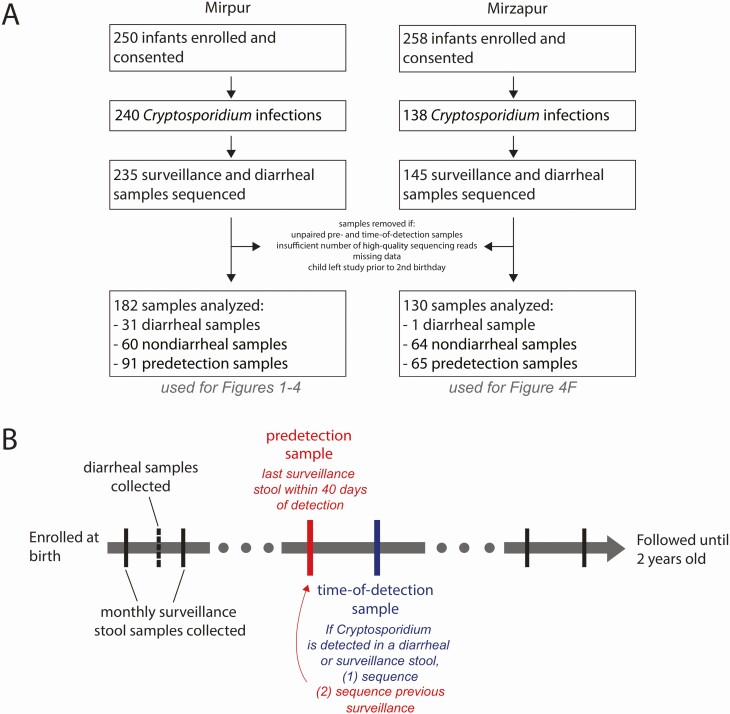
Study design. *A*, Overall cohort design and sample collection. For more information, see [1, 14]. Samples from Mirzapur were only used in post hoc analysis in Figure 4F. *B*, Paired samples were selected to assess *Cryptosporidium*-positive samples (time of detection) and the preceding surveillance sample (predetection). *Cryptosporidium*-positive samples were identified from both monthly surveillance and diarrheal stool samples, generating our subclinical and diarrheal sample groups.

Children were enrolled into a community-based prospective cohort study of enteric infections that was established at the urban and rural Bangladesh sites, Mirpur and Mirzapur, respectively (ClinicalTrials.gov identifier NCT02764918) (Figure 1A) [1, 14]. Stool samples were collected monthly and during diarrheal episodes. Diarrhea was defined as ≥3 loose stools within 24 hours, as reported by the child’s caregiver. Both pan-species and species-specific quantitative polymerase chain reaction assays were used to identify the *Cryptosporidium* species infecting the children (Steiner et al 2018) [1]. If positive samples were collected within an interval of ≤65 days, they were regarded as derived from 1 infection event [1, 14]. In addition to the collection of stool samples, a study database was created containing clinical information on each episode of diarrhea a child experienced, antibiotic consumption, and anthropometric measurements as well as data on the household demographics [1]. A subset of the *Cryptosporidium*-positive and corresponding “predetection” *Cryptosporidium*-negative surveillance samples were analyzed. The data from Mirzapur (Figure 1A) were only included in the post hoc analysis due to the limited amount of information on the antibiotic history of these children, the rarity of diarrheal cases at this site, and the high prevalence of *C. meleagridis* at the site relative to the more common *C. hominis* species detected in Mirpur.

The study was approved by the Ethical and Research Review Committees of the International Centre for Diarrhoeal Disease Research, Bangladesh, and by the Institutional Review Board of the University of Virginia. For each child, informed written consent was obtained from their parent or guardian.

### DNA Extraction

On the day of collection, stool samples were brought to the study clinic and transported to our laboratory at 4°C, where they were aliquoted in DNase- and Rnase-free cryovials for storage at –80°C. For DNA extraction, samples were thawed and 200 mg removed for total nucleic acid extraction (see details in [1]). To verify the extraction protocol, phocine herpesvirus (European Virus Archive Global, Department of Virology, Erasmus Medical Center, Rotterdam, the Netherlands) and bacteriophage MS2 (ATCC 15597B; American Type Culture Collection, Manassas, Virginia) were added into each sample as positive controls.

### 16S Ribosomal Sequencing and Processing

The V4 region of the 16S ribosomal (rRNA) gene was amplified using the previously described phased Illumina-eubacteria primers and protocol from [20, 21] with the minor modification that the Illumina MiSeq version 3 chemistry was used to generate 300-bp paired-end reads. Sequencing was performed by the University of Virginia’s Genome Analysis and Technology Core. Negative controls included extraction blanks throughout the amplification and sequencing process. As positive controls, DNA was extracted from the HM-782D Mock Bacteria Community (ATCC through BEI Resources) and analyzed on each sequencing run (Supplementary Figure 1*A–C*). Additionally, a PhiX DNA library was added at 20% into each sequencing run to increase genetic diversity prior to parallel sequencing in both forward and reverse directions using the Miseq version 3 kit and machine (per the manufacturer’s protocol).

Sequencing produced 48 146 401 reads with a mean of 118 295.8 and median of 121 519 reads per sample (raw reads from Supplementary Figure 1*D*). Sequence adaptors were then removed using Bbtools [22] and primers were removed using CutAdapt [23]; quality-based filtering was performed with DADA2 [24]. Quality filtration reduced the total number of reads to a mean of 59 202.2 reads per sample (Supplementary Figure 1*D*). In brief, reads were removed and trimmed based on overall read quality and base pair quality: forward and reverse reads were trimmed to 250 or 200 bp and removed if there were more than 3 or 6 expected errors, respectively. Reads were also truncated at the first instance of a quality score (Phred or Q score) of ≤2. Next, forward and reverse reads were merged with only 1 mismatch permitted. Last, taxa assignments were made using DADA2’s naive Bayesian classifier method and the Ribosomal Database Project’s Training Set 16 (release 11.5) reference database [24] and reads that did not map to bacteria (including human contaminants, archaea, and mitochondrial or chloroplast DNA) were removed, resulting in a mean of 27 809 reads per sample.

Samples with <10 000 reads and unpaired samples (those with no predetection or time-of-detection sample within 42 days) were removed from consideration; all were subsampled to a uniform depth of 10 000 reads per sample to correct for differences in sequencing depth across samples and to enable the comparison (rather than cataloging) of sequenced taxa among samples [25]. Following these filtration and processing steps, 2953 amplicon sequence variants (ASVs) and 182 stool samples remained in the dataset.

The 182 paired predetection and time-of-detection samples (91 pre- and 91 postdetection), as well as additional positive and negative control samples (amplification blanks) and additional samples that did not pass our selection criteria, were split into 2 sequencing runs to increase the sequencing depth. The first sequencing run included all predetection samples and the second sequencing run included all time-of-detection samples. As an unintentional result of this choice, sequencing batch effects may result in spurious differences between predetection and time-of-detection samples; thus, analyses are focused on symptomatic vs subclinical samples within each time point (ie, within the same sequencing batch).

### Statistical and Machine Learning Analyses

All of the following data processing and statistical analyses were performed in R software [24, 26–29] (see Supplementary Materials for code and software versions). Appropriate statistical tests were selected and are described as introduced throughout the Results.

For machine learning analyses, random forest analysis was used to classify subclinical or diarrheal samples using associated metadata and/or ASV abundances, and the trained models (ie, classifiers) were used to identify individual variables that were important for prediction accuracy [30]. Within a random forest classifier, individual trees were built from subsets of the data and model performance was evaluated by predicting the class of each sample using only the trees in the random forest that were not constructed using that sample (ie, out-of-bag performance). Here, variables were ranked by their effect on classifier certainty, which influenced overall accuracy, using the mean decrease in node impurity (via the Gini coefficient). Variables that maximally split samples by classification group yielded a larger forest-wide node impurity (or evenness of the split); thus, more important variables had a higher mean decrease in node impurity. Analytic code is provided in the Supplementary Materials; analyses and figure generation were performed in R software [29, 31–43].

## RESULTS

### Prevalence of Diarrhea and Antibiotic Use

**Figure 2.**
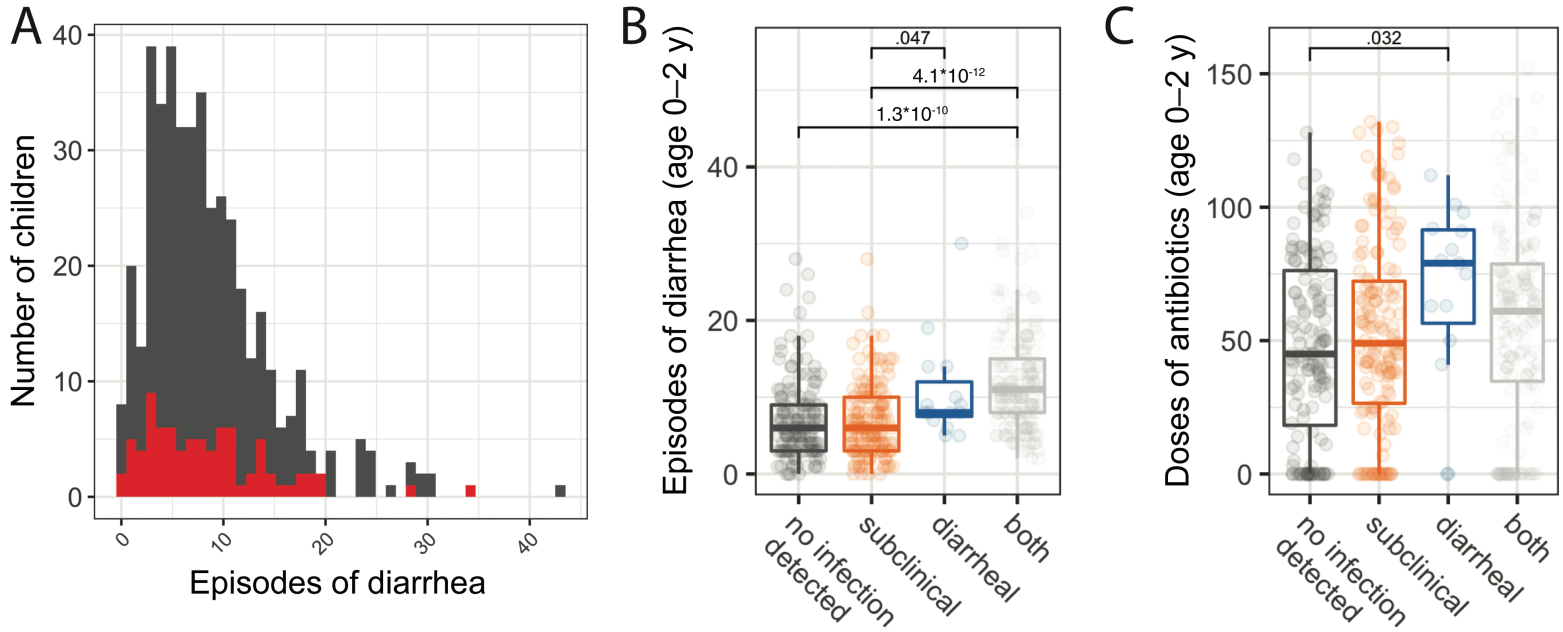
Diarrheal infection and antibiotic treatment were common and heterogenous in infants from Mirpur. *A*, Prevalence of diarrhea. Frequency of diarrheal episodes per child. Full Mirpur cohort is shown in in gray; red subset indicates the children whose samples were used in the microbiome study (and all subsequent figures). *B*, All-cause diarrhea was heterogenous among children with divergent *Cryptosporidium* outcomes. Number of diarrheal events per child based on cumulative *Cryptosporidium* status, both over the first 2 years of life. *C*, Antibiotic usage was heterogenous among children with divergent *Cryptosporidium* outcomes. Number of antibiotic events per child based on cumulative *Cryptosporidium* status, both over the first 2 years of life. Combination therapies were treated as separate doses. For *B* and *C*, the full cohort was used and statistics are shown if significant. For *B* and *C*, each box represents the median (inner line), 25th percentile, and 75th percentile. Upper whiskers extend from the top of the box to the largest value within 1.5 times the interquartile range (distance between 25th and 75th percentile), and the lower whisker extends to the smallest value within 1.5 times the interquartile range. *P* values were generated from a *t* test without multiple testing correction.

Infants were enrolled into a prospective cohort from Mirpur, Dhaka, Bangladesh to study enteric infections (Figure 1A); this cohort was part of a larger assessment of diarrhea in Bangladesh, published previously (Figure 1A) [1]). Each child was monitored by community health workers for enteric disease, including collection of monthly surveillance and diarrheal stool samples during the first 2 years of life. Diarrhea and antibiotic use were common in this cohort (Figure 2A and Supplementary Figure 2), and *Cryptosporidium* species, including *C. hominis* and *C. meleagridis*, were frequently detected during diarrhea (Table 1). These parasites cause both subclinical and overt diarrheal infections [1].

**Table 1.** Sample Summary Statistics for Samples From Mirpur

		Subclinical Infections	Diarrheal Infections	Total
Children				72
	Male/female sex			28/44
	Children with repeat infections in dataset			19
Samples				182
	No. of PD samples	60	31	91
	No. of TOD samples	60	31	91
	Age at collection, d, mean (SD)	362.5 (128.8)	321.3 (136.3)	348.7 (132.1)
	Days between PD and TOD sample^a^, mean (SD)	31.1 (4.6)	19.2 (9.1)	27.0 (8.6)
	Parasite burden at TOD (pan-*Cryptosporidium* qPCR Ct), mean (SD)	28.6 (6.2)	29.9 (7.3)	29.0 (6.6)
	Positive qPCR (for positive samples)	Pan-*Cryptosporidium*: 100%	Pan-*Cryptosporidium*: 100%	Pan-*Cryptosporidium*: 100%
		*C. hominis*: 60%	*C. hominis*: 58%	*C. hominis*: 59%
		*C. meleagridis:* 7%	*C. meleagridis:* 6%	*C. meleagridis:* 7%
	First infection/repeat infection, No.	42/18	28/3	70/21

Abbreviations: Ct, cycle threshold; PD, predetection; qPCR, quantitative polymerase chain reaction; SD, standard deviation; TOD, time of detection.

^a^Statistically different between subclinical and diarrheal infections via *t* test (*P* = 4 × 10^–8^). All other comparisons between clinical types were not significantly different using a *t* test (all comparisons except first vs subsequent infection) or χ ^2^ test (first vs subsequent infection).

Children who had at least 1 symptomatic episode of cryptosporidiosis had more cumulative episodes of diarrhea than children with exclusively subclinical infections or no *Cryptosporidium*-positive stool samples (Figure 2B). Additionally, children with only diarrheal episodes (ie, no observed subclinical cryptosporidiosis) had more frequent exposure to antibiotics than children who had never tested positive for *Cryptosporidium* (Figure 2C). Frequent antibiotic use occurred (Supplementary Figure 2*A*), but there was no difference in antibiotic use during the month prior to infection between children with subclinical or diarrheal infections (Supplementary Figure 2*B*).

### Microbiota Sequencing

Given the difference in all-cause diarrheal frequency between children with subclinical and diarrheal cryptosporidiosis (Figure 2B), we hypothesized that microbiome composition may influence the development of acute symptoms during cryptosporidiosis. 16S rRNA gene sequencing was performed on both the time-of-detection stool samples (*Cryptosporidium* positive, including subclinical and diarrheal) and the corresponding surveillance stool collected immediately prior to the *Cryptosporidium*-positive sample (predetection; Figure 1B) for a subset of children who tested positive for *Cryptosporidium* (Table 1 and Figure 2A). Predetection samples were collected within approximately 1 month of the time-of-detection samples (Table 1).

Sequencing produced 48 146 401 reads with a mean of 118 295.8 and median of 121 519 reads per sample (raw reads from Supplementary Figure 1*D*). Following quality filtration and taxonomy assignment, a mean of 27 809 reads per sample remained, permitting us to subsample reads to a uniform depth of 10 000 reads per sample to correct for differences in sequencing depth across samples.

### Microbiota Diversity

**Figure 3.**
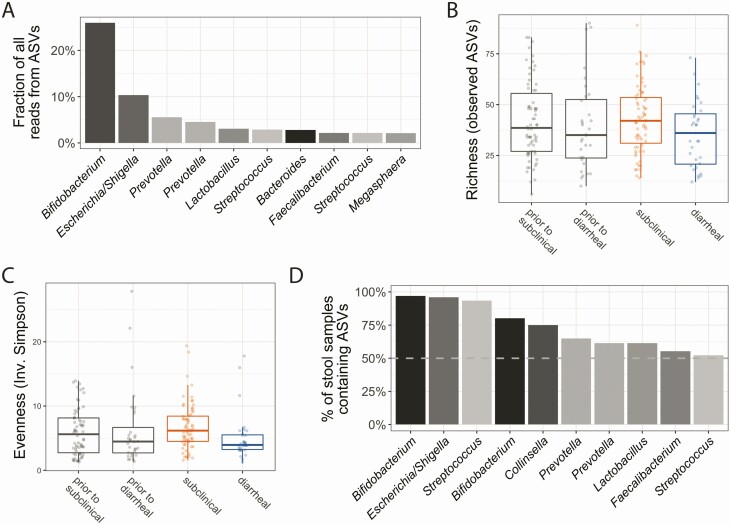
Microbiome samples were highly variable. *A*, Most abundant amplicon sequence variants (ASVs) in the study. Only the top 10 most abundant ASVs are shown; the abundance of these common ASVs per sample is also represented in Supplementary Figure 3. Nearly 25% of all reads were assigned to an ASV in the *Bifidobacterium* genus. *B*, Richness of each sample, or the number of ASVs present in a sample, was not significantly different across sample groups. *B* and *C*, Each box represents the median (inner line), 25th percentile, and 75th percentile. Upper whiskers extend from the top of the box to the largest value within 1.5 times the interquartile range (distance between 25th and 75th percentile), and the lower whisker extends to the smallest value within 1.5 times the interquartile range. *C*, Evenness was also minimally different across sample groups. Evenness is a diversity metric calculated to represent how many different species are present and how well distributed those species are across samples; it is calculated using the inverse Simpson index. No significant differences in evenness was observed among any comparisons of clinical type (2-way analysis of variance with multiple testing correction via Tukey honest significant difference). *D*, Fraction of all samples containing a particular ASV, ordered by from highest to lowest. Very few ASVs were detected in many samples; however, almost all samples contain the most common *Bifidobacterium* ASVs.

Following sequencing, taxonomy was assigned to reads using DADA2. Nearly 25% of reads were assigned to an ASV belonging to the genus *Bifidobacterium* (Figure 3A) that represents a number of functionally diverse species which colonize the infant gastrointestinal tract soon after birth. Microbiota α-diversity measures (richness and evenness) were not statistically significantly different between sample groups (2-way analysis of variance, post hoc testing via Tukey honest significant difference method; significance cutoff of *P* < .05; Figure 3B and 3C). Despite this lack of significance (*P* > .21 for all comparisons), the microbiota of infants who had diarrheal infection was, on average, less diverse than infants with subclinical infection, both prior to and at the time of infection (Figure 3B and 3C). Moreover, this cohort exhibited high interindividual variation as many ASVs were specific to just a few children. Only a few ASVs were found in >50% of samples (Figure 3D).

### Associations Between Diarrheal Symptoms and the Microbiota

**Figure 4.**
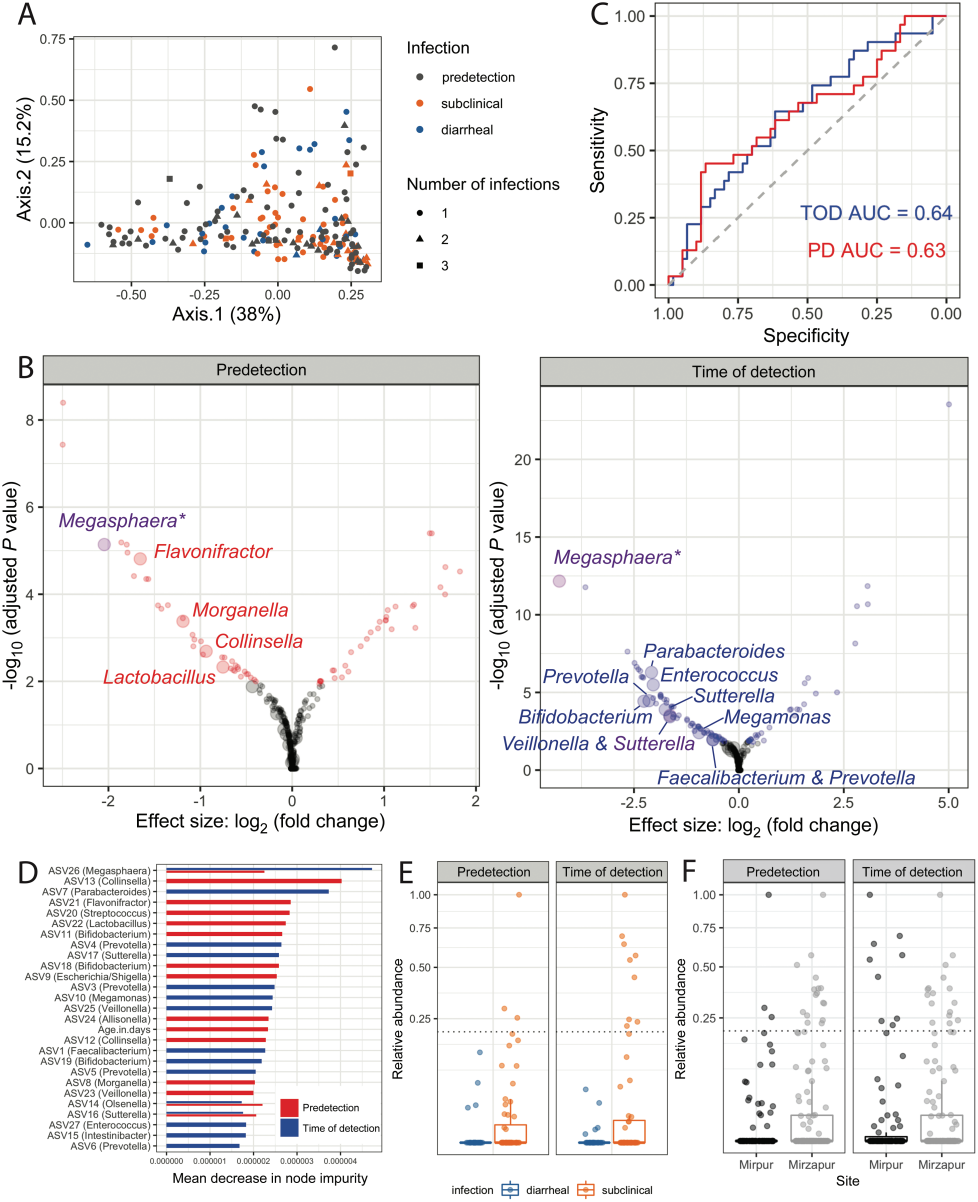
Identifying associations between diarrheal symptoms and the microbiota. *A*, Predetection (PD) and time-of-detection (TOD) sample microbiota were indistinguishable via principal coordinate analysis using a permutational multivariate analysis of variance using distance matrices and a significance cutoff of *P* < .05, as were subclinical and diarrheal *Cryptosporidium*-positive samples. Principal coordinate analysis of amplicon sequence variants (ASV) quantification across all samples using Euclidean distance. *B*, Univariate statistics identifies ASVs associated with symptoms in the PD samples and TOD samples. Statistically significant differential expressed ASVs are colored, whereas gray points represent ASVs that were not different or not significantly different, using DESeq2. Large points indicate ASVs that were also identified as important using random forest classification, whereas small points were not among the top 15 most important variables. Random forest classifiers were built to predict the presence of diarrhea upon *Cryptosporidium* infection. Importantly, purple points represent statistically significant ASVs that were also among the most important variables for classifiers made at both timepoints. *C*, Random forest classifiers were built from the TOD microbiota (blue) or predetection microbiota (red). Area under the curve (AUC), a metric of classifier accuracy, is listed for each classifier. *D*, Most important variables, as ranked by mean decrease in node impurity (or Gini importance), from the PD and TOD classifiers. Important variables were similarly important, within and across models. Of note, age was not an important variable in the TOD classifier. *E*, One ASV assigned to the *Megasphaera* genus was significantly less abundant in diarrheal cases via univariate analyses (at both timepoints) and was among the top 15 most important variables for the classifiers for both timepoints. Relative abundance of each ASV is plotted for each sample, with each box representing the median (inner line), 25th percentile, and 75th percentile. Upper whiskers extend from the top of the box to the largest value within 1.5 times the interquartile range (distance between 25th and 75th percentile), and the lower whisker extends to the smallest value within 1.5 times the interquartile range. *F*, The *Megasphaera* ASV was also more likely to be high-abundance (above dashed line) in samples at the second study site, Mirzapur, where diarrheal cryptosporidiosis was less common when compared to Mirpur; however, environmental factors, including the causal *Cryptosporidium* species, were also different in Mirzapur [1]. Increased *Megasphaera* abundance in Mirzapur may partially explain reduced diarrhea associated with cryptosporidiosis in that community.

To identify compositional differences in the microbiome among sample groups, principal coordinate analysis was performed using the Euclidean distance between samples. Predetection samples overlapped substantially with *Cryptosporidium*-positive samples and, among positive samples, subclinical and diarrheal samples did not separate (permutational multivariate analysis of variance using distance matrices [PERMANOVA]; *P* >* *.05; Figure 4A). Alternative distance metrics, such as Unifrac, also failed to separate sample groups (Supplementary Figure 4*A*). The change in microbiota from predetection to time of detection for each child was similarly variable for both diarrheal and subclinical infections (PERMANOVA; *P* > .05; Supplementary Figure 4*B*).

Given the lack of separation between samples when considering overall microbiome composition, univariate analyses were used to identify individual ASVs that were significantly different between subclinical and diarrheal samples prior to and at the time of infection (Figure 4B). However, univariate statistics rely on assumptions of independence and, thus, may perform poorly with microbiome datasets due to correlations between and statistical interactions among members of the microbiota [44]. To make robust inferences of the importance of individual ASVs, we utilized a univariate approach designed specifically for sparse count data [45], as well as random forest classification to consider interactions among ASVs. Interpreting the results of these 2 approaches together provided a more stringent assessment of ASV importance.

Thus, classification using the random forest models was performed to determine if specific members of the microbiota were predictive of the development of diarrheal symptoms; important variables from the random forest models are highlighted on the volcano plots, which also show the results of univariate statistical tests (Figure 4B and 4C). This machine learning approach was used to prioritize the results generated from univariate statistics. Classifier performance using the predetection or time-of-detection microbiome separately yielded predictive models (area under the curve >0.6 for both prior to and at the time of infection Figure 4C); this performance was similar to the highest-performing classification models across a metanalysis of case-control clinical microbiome studies [46, 47].

Both classifiers supported conclusions drawn by univariate analyses and identified several additional ASVs as important to classify subclinical and diarrheal samples (Figure 4B and 4C). Some important microbes for each classifier were not enriched in either sample group (Figure 4B), suggesting that these ASVs are only important when analyzed in combination with others. Despite the effect of antibiotic treatment on the microbiota [48], the addition of a child’s antibiotic history did not significantly augment classifier performance (Supplementary Figure 5), indicating that there was no interaction between the important ASVs and antibiotic use. The infecting *Cryptosporidium* species (*C. hominis* or *C. meleagridis*) were not important variables in the random forest models, and child age was not an important variable in the time-of-detection model (Figure 4D).

We focused on ASVs that were identified via both the univariate statistics and machine learning approaches. For the predetection timepoint, these prioritized ASVs were assigned to the *Megasphaera*, *Flavonifractor*, *Morganella*, *Collinsella*, and *Lactobacillus* genera; for the time-of-detection timepoint, these included the same *Megasphaera* ASV, as well as ASVs assigned to *Parabacteroides*, *Enterococcus, Prevotella*, *Bifidobacterium*, *Sutterella*, *Veillonella*, *Megamonas*, and *Faecalibacterium* (Figure 4B and 4D). Combinations of ASVs were more predictive of diarrhea than any individual ASV, as evident by the similar Gini importance for all important variables (Figure 3D).

One *Megasphaera* ASV in particular was identified at both timepoints and both analytic approaches (Figure 4B and 4E). This *Megasphaera* ASV also accounted for at least 1% of reads across the entire study (Figure 2A), and was present in 25% of samples (Supplementary Figure 6*A*). This bile acid–resistant species colonizes the small intestines [49], among other sites on the human body [50, 51]. It can therefore be a major component of the microbiome at the site of *Cryptosporidium* parasite colonization. The other ASVs that contributed to model performance were either less abundant or resided predominantly in the large bowel. Interestingly, *Megasphaera* ASVs broadly did not show the same trend as the important individual ASV (Supplementary Figure 6*B*) and were present in 54.9% of samples.

Although there were many environmental differences between the study sites, this ASV was also more likely to be detected at high abundance in our second study site, rural Mirzapur (Figure 4F), despite the observation that *Megasphaera* ASV did not vary with *Cryptosporidium* species (Supplementary Materials). The most common *Cryptosporidium* species at Mirzapur was *C. meleagridis* rather than the *C. hominis* in Mirpur, but *C. meleagridis* has been associated with gastrointestinal disease in other studies and has also been shown to cause diarrhea in a human challenge experiment [52, 53]. Children in Mirzapur were, however, less likely to develop diarrhea upon *Cryptosporidium* infection; 3% of *Cryptosporidium-*positive stools in Mirzapur were diarrheal, compared to 32% in Mirpur [1].

## DISCUSSION

Here, we identified differences in the microbiota composition and in the abundance of an individual ASV, *Megasphaera*, in infants who had either a subclinical or a diarrheal *Cryptosporidium* infection. Fecal samples from 72 *Cryptosporidium*-infected children in Mirpur, Bangladesh, were used to profile the human microbiota during cryptosporidiosis (Table 1 and Figure 1) with 16S rRNA gene sequencing (Figure 3). It is well established that the microbiome shifts with child development [54–56] and that it is highly variable in infants aged <2 years [57–59]. There was also universally frequent antibiotic use and enteric infections in this young population (Table 1, Figure 2C, and Supplementary Figure 2). It was therefore unsurprising that there was a high degree of intersample variability among these infants’ samples (Figure 3A and 3D).

Despite this variation, microbiome composition was predictive of diarrheal symptoms at the time of infection and up to a month prior (Figure 4C). Although individual members of the microbiome were associated with diarrhea (Figure 4B), no single ASV completely explained the clinical type of infection (Figure 4D). This observation is consistent with animal models of infection that have highlighted a complex relationship between the microbiota, host, and parasite [60–62]. For example, previous work found that antibiotics alone did not sensitize immunocompetent mice to infection [18], although certain probiotics [63], antibiotics [64], and deprivation of prebiotics [65] could exacerbate disease severity.

Higher abundance of 1 ASV, *Megasphaera* (class: Clostridia), was associated with subclinical *Cryptosporidium* infection whereas its absence or low abundance was more common in cases of *Cryptosporidium*-associated diarrhea (Figure 4B and 4D). This *Megasphaera* ASV was not associated with antibiotic use in this cohort (Supplementary Figure 5) or all-cause diarrhea (ie, total number of diarrheal episodes; Supplementary Materials). *Megasphaera* species can collocate in the small intestines [49] with *Cryptosporidium*, and they were more frequently observed at high abundance in a community in which diarrhea was rarely seen during cryptosporidiosis (Figure 4F) [1]. *Megasphaera* are known to synthesize short-chain fatty acids [66], compounds that regulate the intestinal homeostasis [67], impact the host immune response [68], and modulate osmotic diarrhea [69]. Interestingly, *Megasphaera elsdenii* is used as a probiotic in veterinary medicine to treat diet-induced metabolic acidosis because of the bacteria’s ability to convert lactate (a key acidic metabolite responsible for acidosis) to short-chain fatty acids [70]. This ability of *Megasphaera* to produce short-chain fatty acids or to modulate the host’s immune system through other mechanisms may be protective in attenuating disease outcome during *Cryptosporidium* infection. Alternatively, *Megasphaera* may be a biomarker for another microbiome- or immune-mediated mechanism of protection from diarrhea.

Limitations of this study include the wide age range of children enrolled in this study, the microbial diversity of samples, widespread antibiotic use and infections, and the unknown generalizability to global populations. In addition, technical limitations include moderate sample size, the fact that time-of-detection and predetection samples were sequenced separately, and the need for read count normalization due to the variable sequencing depth across samples.

In sum, the microbiome was predictive of *Cryptosporidium* diarrhea both prior to and at the time of infection. Low abundance of 1 member of the microbiome, *Megasphaera*, was associated with diarrheal symptoms. There is currently no effective drug for treating *Cryptosporidium* diarrhea in children, and modulating members of the microbiota such as *Megasphaera* may be an appealing prevention strategy.

## Supplementary Data

Supplementary materials are available at *Clinical Infectious Diseases* online. Consisting of data provided by the authors to benefit the reader, the posted materials are not copyedited and are the sole responsibility of the authors, so questions or comments should be addressed to the corresponding author.

ciab207_suppl_Supplementary_Figure_1

ciab207_suppl_Supplementary_Figure_2

ciab207_suppl_Supplementary_Figure_3

ciab207_suppl_Supplementary_Figure_4

ciab207_suppl_Supplementary_Figure_5

ciab207_suppl_Supplementary_Figure_6

ciab207_suppl_Supplementary_Material

## Notes

***Author contributions*.** M. A. C. and C. A. G. conceived of the study and W. A. P. led the study. R. H. and A. S. G. F. founded the birth cohort and directed the Bangladesh studies. G. L. M. and M. A. C. conceived of the analyses. Field work and data collection at the International Centre for Diarrhoeal Disease Research, Bangladesh (icddr,b) were performed by M. A. and M. K., with A. S. G. F. and R. H. providing supervision. DNA extraction and 16S rRNA library preparation were performed by M. J. U., and U. N. oversaw the clinical database. G. L. M. performed some preliminary microbiome sequence processing and machine learning analyses, with supervision by J. P. Final analyses and drafting of the manuscript were performed by M. A. C. All authors edited and approved the final manuscript.

***Acknowledgments.*** The authors thank the children and parents from the icddr,b study sites as well as the field workers, physicians, scientists, and staff of the Emerging Infectious Diseases Division of icddr,b for their key contributions to this research. The authors also thank the members of the Petri lab group for their feedback, especially Jeff Donowitz, Kevin Steiner, Chelsea Marie, and Barbara Mann, and the University of Virginia Genome Analysis and Technology Core for conducting the sequencing for this study.

***Disclaimer.*** The funders had no role in study design, data collection and analysis, or decision to submit for publication.

***Financial support.*** This work was supported by the National Institutes of Health (grant numbers R01 AI043596 to W. A. P. and C. A. G.; T32 LM012416 to J. P. and G. L. M.; and R21 142656 to C. A. G.); the University of Virginia (Engineering-in-Medicine Seed funding to M. A. C., J. P., and W. A. P.); the Bill & Melinda Gates Foundation (grant number OPP1100514); and the PhRMA Foundation (Postdoctoral Fellowship in Translational Medicine and Therapeutics to M. A. C.). The governments of Bangladesh, Canada, Sweden, and the United Kingdom provide core support to icddr,b.

***Potential conflicts of interest.*** W. A. P. acts as a consultant for TechLab Inc, which produces diagnostics for cryptosporidiosis. All other authors report no potential conflicts of interest. All authors have submitted the ICMJE Form for Disclosure of Potential Conflicts of Interest. Conflicts that the editors consider relevant to the content of the manuscript have been disclosed.
