# Supplementary figures and images for: Megasphaera in the Stool Microbiota Is Negatively Associated With Diarrheal Cryptosporidiosis
Source: Clin Infect Dis. 2021 May 4;73(6):e1242–51. doi: 10.1093/cid/ciab207 (PMC8442784; doi:10.1093/cid/ciab207)

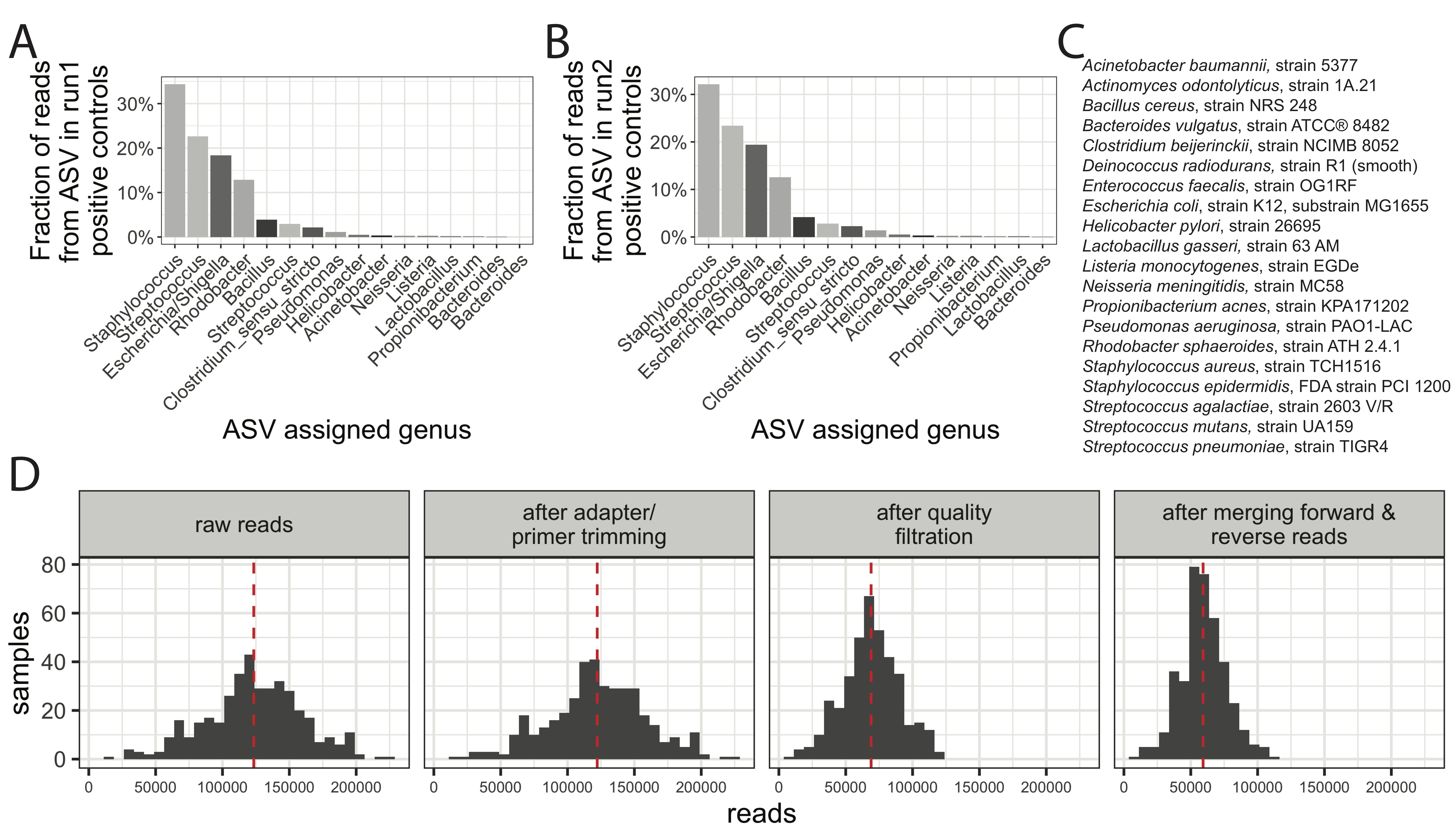

Supplement: ciab207_suppl_Supplementary_Figure_1 [file ciab207_suppl_supplementary_figure_1.jpeg]

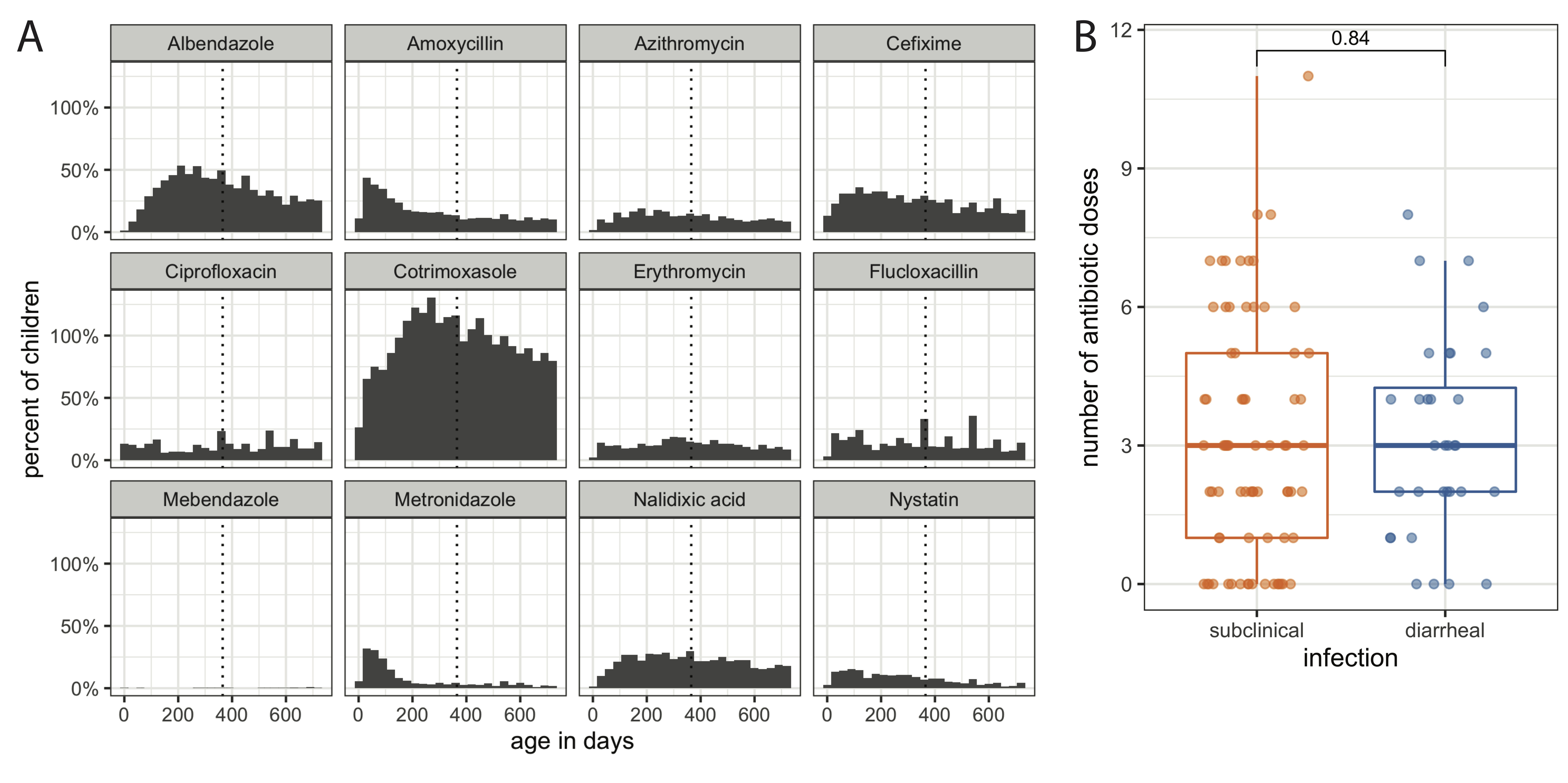

Supplement: ciab207_suppl_Supplementary_Figure_2 [file ciab207_suppl_supplementary_figure_2.jpeg]

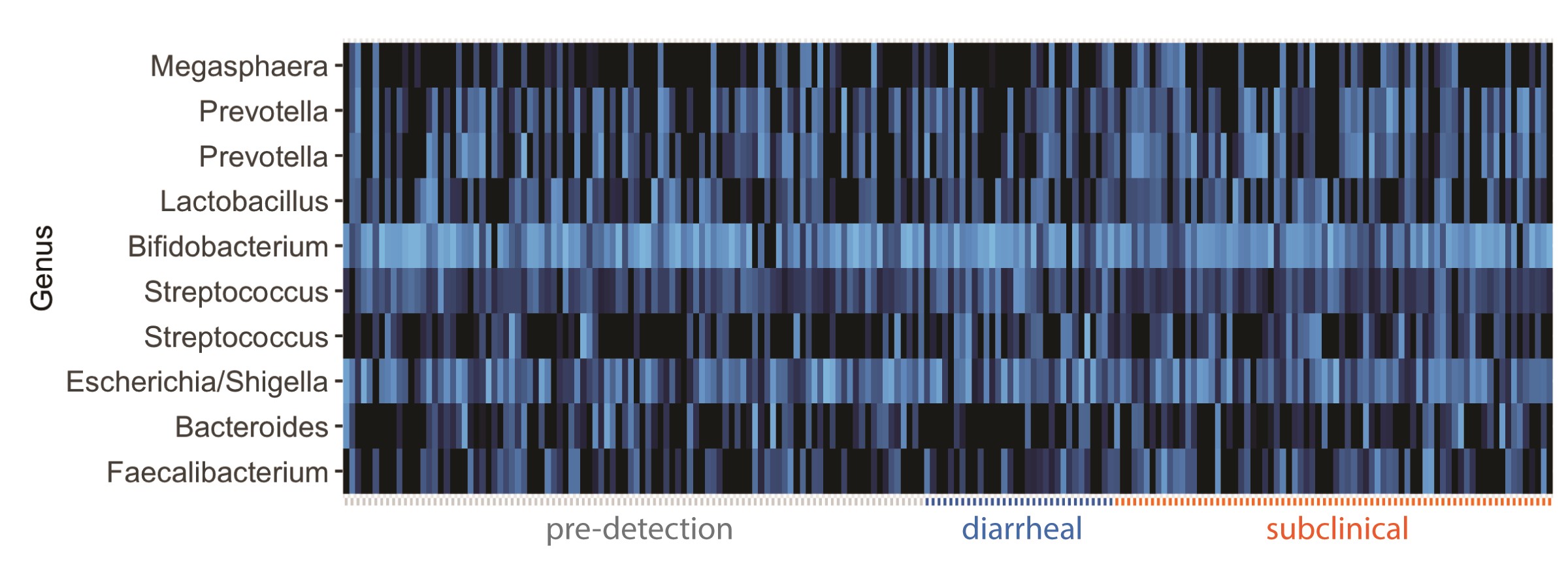

Supplement: ciab207_suppl_Supplementary_Figure_3 [file ciab207_suppl_supplementary_figure_3.jpeg]

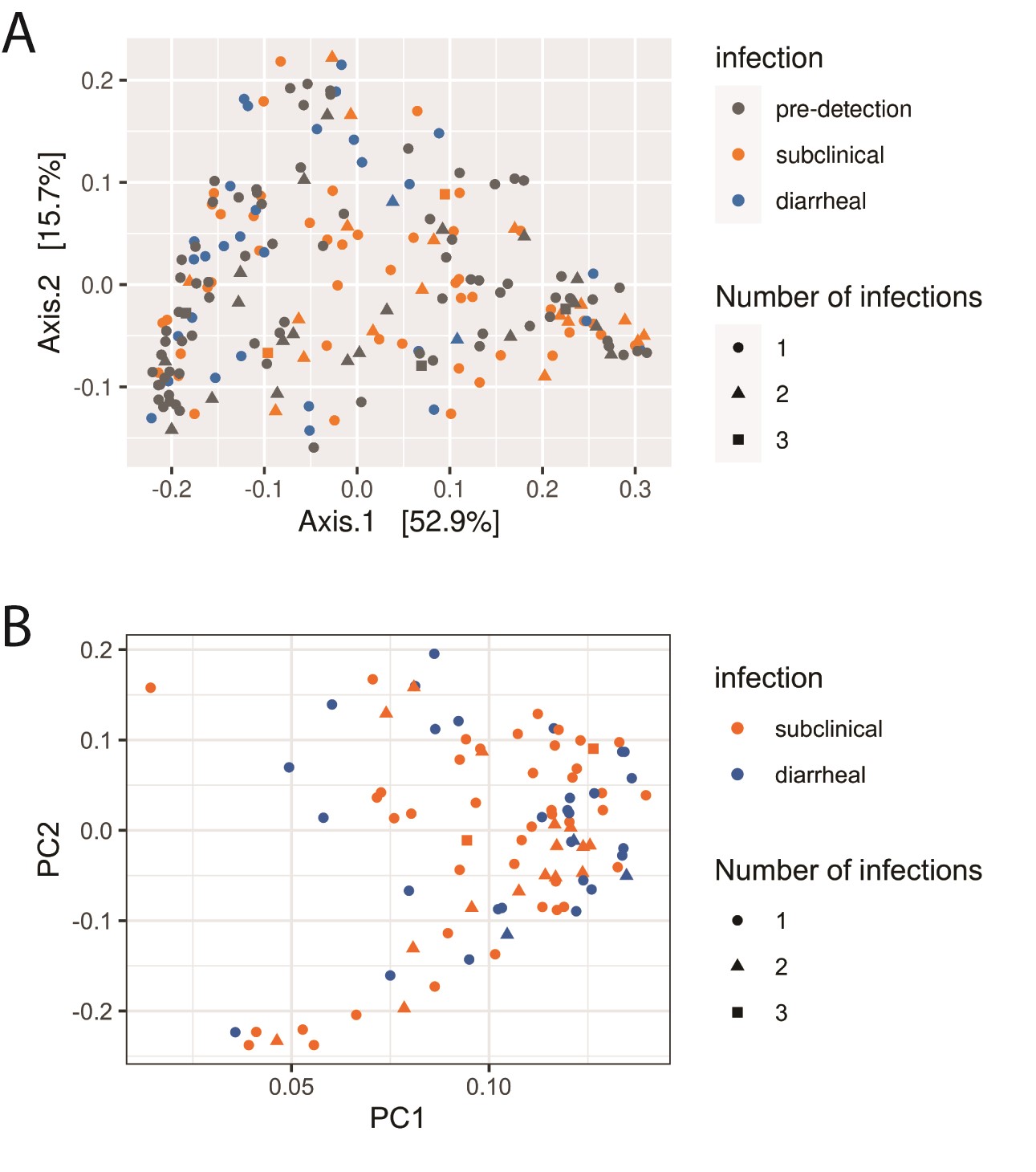

Supplement: ciab207_suppl_Supplementary_Figure_4 [file ciab207_suppl_supplementary_figure_4.jpeg]

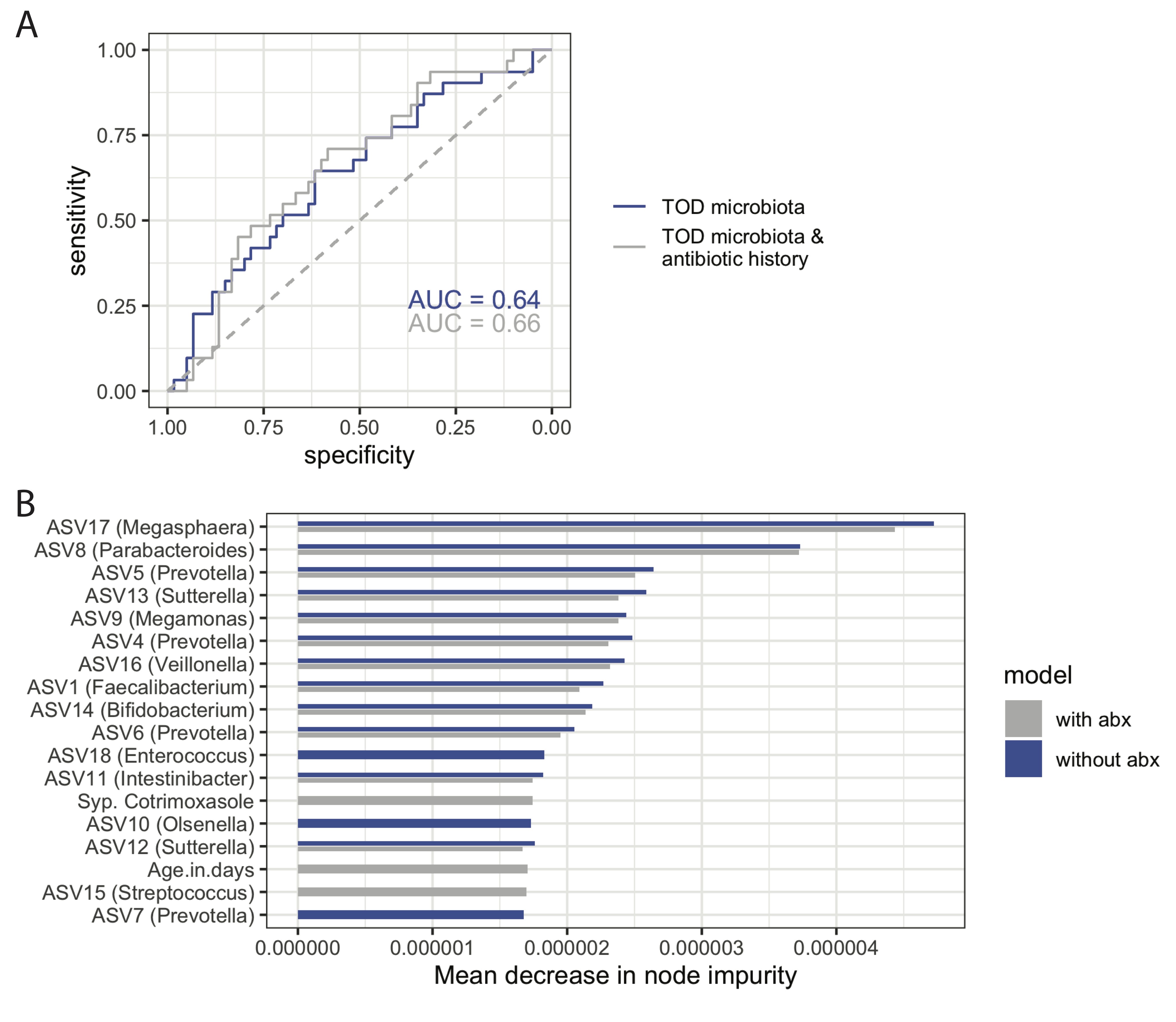

Supplement: ciab207_suppl_Supplementary_Figure_5 [file ciab207_suppl_supplementary_figure_5.jpeg]

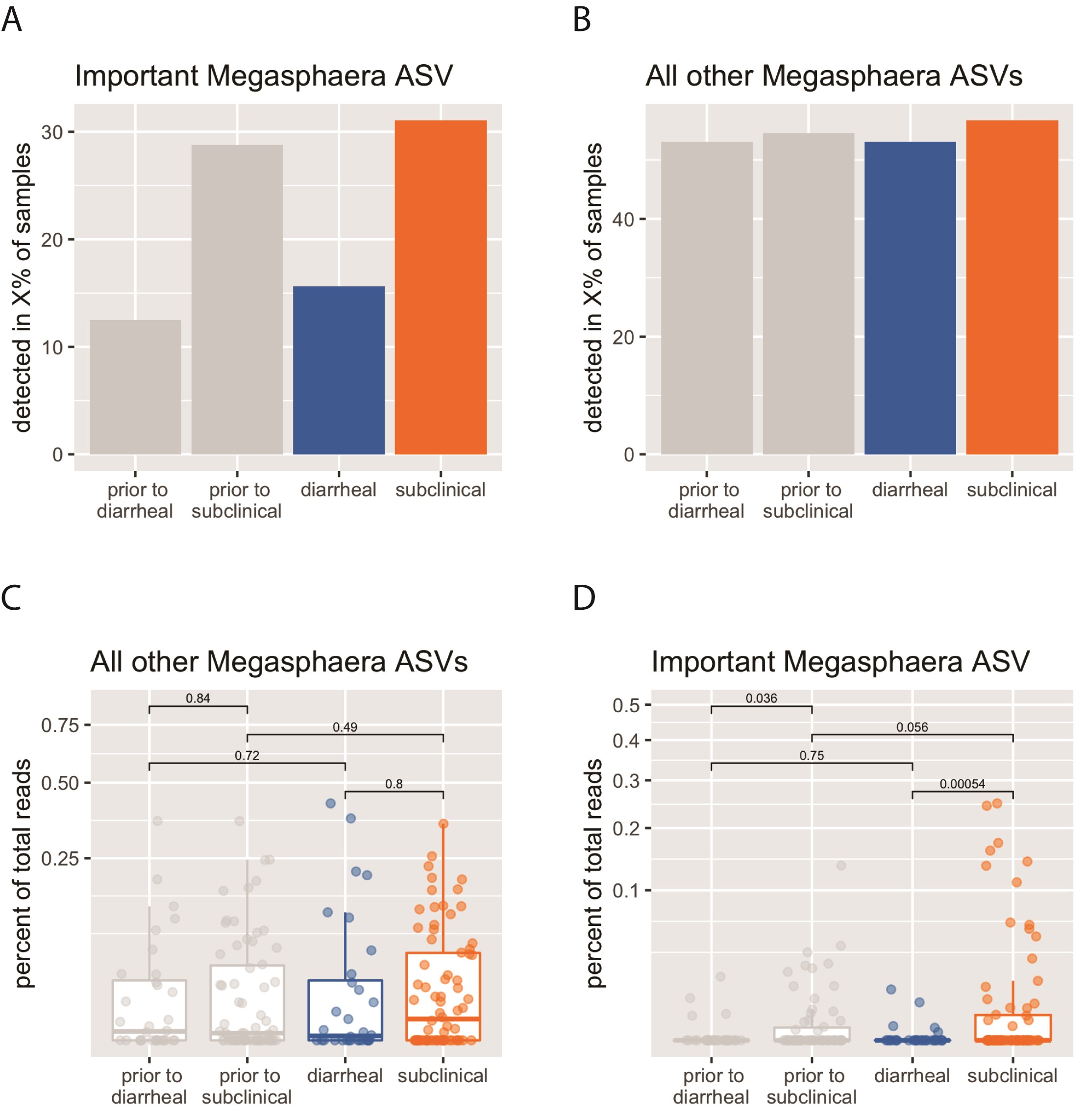

Supplement: ciab207_suppl_Supplementary_Figure_6 [file ciab207_suppl_supplementary_figure_6.jpeg]
