# Supplementary material for: Megasphaera in the Stool Microbiota Is Negatively Associated With Diarrheal Cryptosporidiosis
Source: Clin Infect Dis. 2021 May 4;73(6):e1242–51. doi: 10.1093/cid/ciab207 (PMC8442784; doi:10.1093/cid/ciab207)
Supplement: ciab207_suppl_Supplementary_Material [file ciab207_suppl_supplementary_material.docx]

Supplementary materials, particularly analytic code, are available at https://github.com/maureencarey/cryptosporidium_microbiome and archived as DOI: 10.5281/zenodo.4632159. Select clinical metadata are available at the National Center for Biotechnology Information Database of Genotypes and Phenotypes (dbGaP) under accession number phs001665.v2.p1. The data for this study are collected as a substudy of dbGaP phs001475.v2.p1. Raw sequence data generated for this study will be available in the Sequence Read Archive upon publication. Code for analysis is available at https://github.com/maureencarey/cryptosporidium_microbiome. Per the consent of the parents and guardians of the children in this study, all other deidentified data may be available upon request.
